# Reviving Hope: A Comprehensive Review of Post-resuscitation Care in Pediatric ICUs After Cardiac Arrest

**DOI:** 10.7759/cureus.50565

**Published:** 2023-12-15

**Authors:** Sri Sita Naga Sai Priya K, Amar Taksande, Revat J Meshram

**Affiliations:** 1 Pediatrics, Jawaharlal Nehru Medical College, Datta Meghe Institute of Higher Education and Research, Wardha, IND

**Keywords:** rehabilitation strategies, multidisciplinary collaboration, neurological considerations, pediatric intensive care units, post-resuscitation care, pediatric cardiac arrest

## Abstract

This comprehensive review thoroughly examines post-resuscitation care in pediatric ICUs (PICUs) following cardiac arrest. The analysis encompasses adherence to resuscitation guidelines, advances in therapeutic interventions, and the nuanced management of neurological, cardiovascular, and respiratory considerations during the immediate post-resuscitation phase. Delving into the complexities of long-term outcomes, cognitive and developmental considerations, and rehabilitation strategies, the review emphasizes the importance of family-centered care for pediatric survivors. A call to action is presented, urging continuous education, research initiatives, and quality improvement efforts alongside strengthened multidisciplinary collaboration and advocacy for public awareness. Through implementing these principles, healthcare providers and systems can collectively contribute to ongoing advancements in pediatric post-resuscitation care, ultimately improving outcomes and fostering a culture of excellence in pediatric critical care.

## Introduction and background

Cardiac arrest in pediatric patients is a critical medical emergency that demands immediate attention and specialized care. The significance of pediatric cardiac arrest lies not only in its relatively rare occurrence but also in the profound impact it can have on the affected children and their families. Advances in resuscitation techniques over the years have played a pivotal role in improving outcomes, offering a glimmer of hope where once the prognosis seemed bleak [1]. Pediatric cardiac arrest represents a distressing and life-threatening event, necessitating an urgent and coordinated medical response. Despite its infrequency compared to adult cardiac arrest, the unique challenges posed by pediatric cases make them particularly complex. Children can experience cardiac arrest due to various underlying conditions, including congenital heart diseases, respiratory illnesses, and traumatic injuries. The consequences of pediatric cardiac arrest extend beyond the immediate medical implications, affecting the long-term well-being and quality of life of survivors [2].

Understanding the distinct nature of pediatric cardiac arrest is crucial for healthcare providers, as it requires tailored approaches to resuscitation, post-resuscitation care, and rehabilitation. Factors such as age, weight, and underlying health conditions contribute to the complexity of managing these cases. Hence, exploring the latest advancements in resuscitation techniques becomes imperative for enhancing the overall care and outcomes of pediatric patients facing cardiac arrest [3]. Over the years, there have been remarkable strides in resuscitation, driven by advancements in medical science and technology and a deeper understanding of the physiological responses during cardiac arrest. These innovations have significantly influenced the outcomes of both adult and pediatric resuscitation efforts. Key developments include improvements in cardiopulmonary resuscitation (CPR) techniques, the widespread availability of automated external defibrillators (AEDs), and the incorporation of evidence-based guidelines into clinical practice [4]. In the context of pediatric resuscitation, specialized considerations such as age-appropriate defibrillation energy levels, medication dosages, and airway management techniques have evolved to address the unique needs of children. The integration of simulation training and the emphasis on a multidisciplinary approach has further contributed to enhancing the preparedness of healthcare teams in managing pediatric cardiac arrest scenarios [5].

This review aims to comprehensively explore the landscape of post-resuscitation care in pediatric ICUs (PICUs) following cardiac arrest. By synthesizing the existing knowledge on this critical topic, we seek to provide healthcare professionals, researchers, and policymakers with a holistic understanding of the challenges and advancements in managing pediatric patients after successful resuscitation.

## Review

Epidemiology of pediatric cardiac arrest

The incidence rates, common causes, and demographic considerations of pediatric cardiac arrest are essential factors in understanding the epidemiology of this critical condition. The overall incidence rate for out-of-hospital cardiac arrest (OHCA) in the United States is 8.3 per 100,000 person-years, with a survival to hospital discharge rate of 11.3% [6]. Pediatric OHCA rates are reported to be 6-10 per 100,000 person-years, with only about 10% of patients surviving hospital discharge [7]. In-hospital cardiac arrest (IHCA) occurs in 2.6%-6% of children with cardiac disease [8]. Pre-arrest characteristics, including age and preexisting chronic conditions, are associated with varying cardiac arrest risk; infants and children with acquired and congenital heart disease are at higher risk [7].

Children with cardiac disease suffer cardiac arrest at rates of 2.6% to 6%, with corresponding survival ranging from 32-50.6% [8]. The survival rate for pediatric IHCA is higher, with 44-52% of patients surviving hospital discharge [7]. The incidence of pediatric cardiac arrest is relatively low, but the survival rates vary depending on whether the arrest occurs in the out-of-hospital or in-hospital setting. Pre-existing chronic conditions and congenital heart disease are significant risk factors for pediatric cardiac arrest.

Resuscitation techniques in pediatric cardiac arrest

Overview of Current Guidelines

Guidelines for pediatric cardiac arrest emphasize a systematic and evidence-based approach to resuscitation. Organizations such as the American Heart Association (AHA) and the European Resuscitation Council (ERC) regularly update and disseminate these guidelines to healthcare providers. The recommendations encompass various aspects, including the recognition of cardiac arrest, the initiation of CPR, and the use of interventions such as defibrillation and medications [9]. Key components of the guidelines address the importance of high-quality CPR, the significance of early defibrillation in shockable rhythms, and the proper sequence of interventions in both basic and advanced life support. Understanding and adherence to these guidelines are critical for healthcare professionals to optimize the chances of successful resuscitation and improve overall patient outcomes [9].

Advances in CPR

Recent years have witnessed notable advancements in CPR techniques, driven by a deeper understanding of the physiological mechanisms during cardiac arrest. High-quality CPR involves adequate chest compressions, proper ventilation, and minimizing interruptions to maintain perfusion. Innovations include real-time feedback devices that assist healthcare providers in optimizing compression depth, rate, and recoil [10]. Moreover, the concept of pit crew CPR, emphasizing a coordinated and efficient team approach, has gained prominence. This approach recognizes the importance of communication, role clarity, and swift transitions between chest compressors to minimize interruptions. Integrating technology, simulation training, and ongoing education further enhances healthcare teams' proficiency in performing high-quality CPR, especially in the dynamic and stressful environment of pediatric cardiac arrest [11].

Role of Automated External Defibrillators (AEDs)

AEDs play a crucial role in the early management of pediatric cardiac arrest, particularly in cases involving shockable rhythms such as ventricular fibrillation or pulseless ventricular tachycardia. AEDs are designed to be user-friendly and can be employed by bystanders, including non-medical personnel. Their widespread availability in public spaces, schools, and healthcare settings has contributed significantly to improving the accessibility of early defibrillation [12]. In pediatric cases, the appropriate use of AEDs involves the consideration of pediatric pads or dose attenuators to deliver a shock suitable for the child's size and age. Integrating AED training in educational curricula and public awareness campaigns further enhances the community's ability to respond effectively to pediatric cardiac arrest scenarios [12].

Importance of Early Recognition and Intervention

Early recognition of pediatric cardiac arrest is a critical determinant of successful resuscitation. Healthcare providers, caregivers, and bystanders must be trained to promptly identify signs of cardiac arrest, including the absence of a palpable pulse, unresponsiveness, and abnormal breathing patterns. Timely activation of the emergency medical system (EMS) and initiation of CPR are paramount in preserving brain and organ function during the critical minutes following cardiac arrest [13]. The window of opportunity for successful intervention narrows rapidly, emphasizing the need for a swift and coordinated response. Public awareness campaigns, community training initiatives, and integration of basic life support skills into educational curricula contribute to empowering individuals to recognize and respond to pediatric cardiac arrest effectively. Early intervention not only improves the likelihood of successful resuscitation but also plays a pivotal role in shaping long-term outcomes for pediatric patients who experience cardiac arrest [13].

Immediate post-resuscitation phase

Stabilization and Monitoring

Stabilization: Following spontaneous circulation return (ROSC), stabilization is the initial priority in post-resuscitation care for pediatric patients. This critical phase involves addressing immediate threats to life to ensure the patient's physiological stability. Key components include securing the airway to facilitate adequate ventilation, ensuring optimal oxygenation levels, and promptly addressing any underlying factors that may have precipitated the cardiac arrest. By swiftly addressing these aspects, healthcare providers aim to establish a foundation for further interventions and prevent the escalation of potential complications [14].

Monitoring: Continuous monitoring is paramount to closely track the pediatric patient's physiological parameters during the immediate post-resuscitation phase. Vital signs, including heart rate, blood pressure, respiratory rate, and oxygen saturation, are meticulously observed. Continuous ECG monitoring is employed to detect abnormalities in cardiac rhythm or signs of cardiac instability. This real-time surveillance enables healthcare teams to promptly identify and respond to changes, allowing for targeted interventions and adjustments to the ongoing management plan [15].

Post-cardiac arrest syndrome: Recognition and management of post-cardiac arrest syndrome are central in the immediate post-resuscitation phase. This syndrome encompasses a complex interplay of systemic ischemia-reperfusion response, myocardial dysfunction, and neurological injury. Early identification of the components of post-cardiac arrest syndrome is crucial for tailoring interventions to mitigate their impact on overall patient outcomes. This may involve strategies to optimize oxygen delivery to tissues, stabilize cardiac function, and prevent or minimize neurological injury. By addressing the multifaceted nature of post-cardiac arrest syndrome, healthcare providers aim to enhance the chances of a favorable recovery and improve the overall prognosis for pediatric patients who have undergone resuscitation [16].

Neurological Assessment

Immediate evaluation: Neurological assessment takes precedence immediately after stabilization in post-resuscitation care for pediatric patients. The Glasgow Coma Scale (GCS) or age-appropriate alternatives are pivotal tools to gauge the patient's level of consciousness. This swift and systematic evaluation enables healthcare providers to ascertain the patient's neurological status rapidly. The findings from this initial assessment guide the subsequent course of interventions and play a crucial role in prognostication. Early identification of neurological deficits lays the foundation for targeted and individualized neuroprotective strategies [17].

Tools for assessment: The arsenal of assessment tools extends beyond traditional measures, incorporating advanced monitoring techniques to glean real-time insights into cerebral function. Continuous electroencephalography (EEG) provides ongoing monitoring of brain electrical activity, aiding in detecting seizure activity or other abnormal patterns. Near-infrared spectroscopy (NIRS) offers a non-invasive means to assess cerebral oxygenation and perfusion, contributing valuable information for managing potential neurological complications. These sophisticated tools enhance the precision of neurological assessment, enabling healthcare teams to tailor interventions based on dynamic and specific neurophysiological data [18].

Therapeutic hypothermia: Embracing therapeutic hypothermia represents a well-established and effective intervention to mitigate neurological injury in the immediate post-resuscitation phase. This evidence-based approach involves carefully lowering the patient's body temperature to a predefined target range. By inducing hypothermia, healthcare providers aim to confer neuroprotection and reduce the risk of secondary brain injury. This therapeutic strategy is particularly relevant in the context of post-cardiac arrest care, where the potential for neurological compromise is heightened. The meticulous application of therapeutic hypothermia underscores a proactive approach to preserving neurological function and improving overall outcomes in pediatric patients who have experienced cardiac arrest [19].

Hemodynamic Management

Fluid resuscitation: The cornerstone of hemodynamic management in the post-resuscitation phase is adequate fluid resuscitation. Maintaining hemodynamic stability is contingent on carefully titrated fluid administration. Close monitoring of central venous pressure (CVP) or other relevant hemodynamic parameters guides healthcare providers in assessing fluid responsiveness and tailoring fluid management strategies. This meticulous approach optimizes intravascular volume, prevents hypovolemia, and supports overall cardiovascular function [20].

Inotropic support: In persistent hemodynamic instability, initiating inotropic and vasopressor support becomes crucial to post-resuscitation care. Agents such as dopamine, epinephrine, and other inotropes are employed and titrated to address specific needs, optimizing cardiac output and blood pressure. The judicious use of inotropic support is guided by continuous monitoring of hemodynamic parameters and the patient's response to intervention. This dynamic approach ensures a tailored, patient-centered strategy to enhance myocardial contractility and systemic perfusion [21].

Goal-directed therapy: Hemodynamic management extends beyond immediate stabilization to incorporate goal-directed therapy. This proactive approach involves setting and achieving specific targets for perfusion, oxygen delivery, and other relevant hemodynamic parameters. Individualized for each patient, goal-directed therapy ensures that the unique responses and requirements of the pediatric patient guide interventions. Regular reassessment and adjustments to therapeutic strategies contribute to the precision of care, aiming for optimal tissue perfusion and overall hemodynamic balance. The implementation of goal-directed therapy reflects a nuanced and adaptive approach to post-resuscitation hemodynamic management [22].

Respiratory Support

Mechanical ventilation: The initiation and management of mechanical ventilation represent pivotal aspects of post-resuscitation care for many pediatric patients. Following cardiac arrest, respiratory function may be compromised, necessitating the provision of mechanical support. Ventilator settings, including tidal volume, respiratory rate, and positive end-expiratory pressure (PEEP), are meticulously adjusted to achieve a delicate balance. The primary goal is to optimize oxygenation and ventilation while minimizing the risk of ventilator-induced lung injury. This involves considering factors such as lung compliance, airway resistance, and the overall respiratory status of the patient [23].

Oxygenation targets: Oxygenation targets play a crucial role in post-resuscitation care, and it is essential to consider factors such as permissive hypercapnia and the role of CO2. Notably, the data pertaining to pediatric arrests has a specific focus on cardiac patients, particularly those with pulmonary hypertension [24]. Continuous monitoring of arterial blood gases and oxygen saturation provides real-time insights into the patient's oxygen status. This information serves as a guide for healthcare providers to make necessary adjustments to oxygen delivery and ventilation strategies. The goal is to ensure that oxygen levels align with the unique needs of pediatric patients. Striking the right balance is paramount, as it helps avoid both hypoxia and hyperoxia, given the potential adverse effects associated with oxygen imbalances. This vigilant approach to oxygenation targets is critical in preventing complications and optimizing respiratory support during the post-cardiac arrest phase [24].

Temperature Management

Normothermia maintenance: Temperature management emerges as a pivotal consideration in post-resuscitation care, with a primary goal of maintaining or achieving targeted therapeutic hypothermia. The choice between normothermia and therapeutic hypothermia is guided by the patient's clinical condition and established protocols. In cases where therapeutic hypothermia is indicated, external cooling devices or other targeted measures are employed to carefully lower the patient's body temperature to a predefined therapeutic range. Conversely, for patients in whom normothermia is preferred, proactive warming measures are implemented to prevent hypothermia and support overall physiological stability [25].

Prevention of hyperthermia: Actively preventing hyperthermia is integral to temperature management post-resuscitation, mainly due to its potential to exacerbate neurological injury. Continuous temperature monitoring ensures timely detection of deviations from the targeted range, prompting swift interventions to prevent hyperthermia. These interventions may include cooling or warming measures adjustments, pharmacological interventions, or other methods tailored to the individual patient's needs. By maintaining a meticulous approach to temperature regulation, healthcare providers aim to create an environment conducive to optimal recovery, minimizing the risk of complications associated with temperature fluctuations in the post-cardiac arrest phase [26].

Neurological considerations

Brain Injury After Cardiac Arrest

Ischemia-reperfusion injury: The intricate process of cardiac arrest and subsequent resuscitation introduces a unique set of challenges, prominently featuring ischemia-reperfusion injury. The initial deprivation of oxygen and nutrients during cardiac arrest sets the stage for a complex sequence of events. While vital for survival, the subsequent restoration of blood flow initiates a cascade that can lead to cellular damage and, critically, brain injury. Understanding the nuances of ischemia-reperfusion injury is pivotal in devising strategies to minimize its impact and optimize neurological outcomes in the aftermath of pediatric cardiac arrest [27].

Global hypoxic-ischemic injury: Pediatric patients, due to their developmental vulnerabilities, are particularly susceptible to global hypoxic-ischemic injury, a condition that can result in varying degrees of neurological impairment. Several factors, including the duration of cardiac arrest, the effectiveness of resuscitation efforts, and the underlying cause of the arrest influence the severity of the resultant brain injury. Recognizing the unique susceptibility of pediatric patients to global hypoxic-ischemic injury underscores the importance of tailored and vigilant post-resuscitation care to mitigate neurological consequences [28].

Secondary brain injury: In the post-resuscitation phase, efforts are concentrated on preventing or mitigating secondary brain injury. Beyond the immediate challenges posed by ischemia-reperfusion injury, factors such as fluctuations in blood pressure, oxygen levels, and body temperature become critical determinants of ongoing neurological damage. Careful management and monitoring of these variables are imperative to prevent the exacerbation of injury and support the delicate balance required for optimal neurological recovery. The emphasis on preventing secondary brain injury reflects a proactive and holistic approach to post-cardiac arrest care, aiming to safeguard against additional insults that could compromise neurological outcomes in pediatric patients [29].

Tools for Neurological Assessment

GCS: The GCS stands as a foundational tool for neurological assessment, and for younger patients, age-appropriate alternatives are employed. This standardized and widely utilized scale provides a systematic method for evaluating the level of consciousness based on three key components: eye response, verbal response, and motor response. The GCS serves as a vital metric for gauging neurological function and is instrumental in the initial assessment of pediatric patients' post-cardiac arrest, providing valuable information to guide subsequent interventions and prognostication [17].

Continuous EEG monitoring: Continuous EEG monitoring emerges as a dynamic and essential tool for real-time insights into brain activity during post-cardiac arrest. This continuous surveillance is particularly valuable for detecting seizures, a common occurrence in the aftermath of cardiac arrest. Timely identification of seizures through continuous EEG monitoring allows for prompt intervention, preventing further neurological injury. The nuanced information provided by continuous EEG monitoring contributes to a more comprehensive understanding of the evolving neurophysiological status of pediatric patients, guiding healthcare providers in tailoring interventions to optimize neurological outcomes [30].

NIRS: NIRS represents a non-invasive and valuable tool employed in post-resuscitation care to monitor cerebral oxygenation levels. By utilizing near-infrared light, NIRS enables the assessment of oxygen delivery to the brain, offering insights into the adequacy of cerebral perfusion. Continuous monitoring with NIRS guides healthcare providers in real time, facilitating interventions to optimize cerebral oxygenation and prevent hypoxic events. This technology adds a layer of precision to neurological assessment, allowing for targeted and proactive measures to support optimal brain function in the critical post-cardiac arrest phase of pediatric care [31].

Therapeutic Hypothermia and Its Effectiveness

The rationale for therapeutic hypothermia: Therapeutic hypothermia, a deliberate lowering of the body temperature, is grounded in the rationale of mitigating neurological injury following events such as cardiac arrest. This intervention is founded on the understanding that hypothermia can induce several neuroprotective mechanisms. By reducing the body's metabolic demands, therapeutic hypothermia minimizes the potential for cellular damage during periods of reduced oxygen supply. Additionally, hypothermia has anti-inflammatory properties, attenuating the inflammatory response that often accompanies neurological insults. Furthermore, by limiting the extent of secondary brain injury, therapeutic hypothermia emerges as a crucial strategy to optimize neurological outcomes in the aftermath of critical events [32].

Targeted temperature management: Implementing therapeutic hypothermia involves precisely controlling the patient's body temperature within a targeted and predefined range. This meticulous management is typically achieved through specialized cooling devices, such as cooling blankets or intravascular cooling catheters. Close monitoring of temperature parameters ensures that the targeted range is maintained consistently throughout the intervention. The duration and depth of hypothermia are crucial aspects determined based on individual patient factors, such as age, underlying health conditions, and the nature of the neurological insult. The careful orchestration of targeted temperature management reflects a commitment to optimizing the therapeutic effects of hypothermia while avoiding potential complications [25].

Effectiveness in pediatric patients: While therapeutic hypothermia has demonstrated efficacy in improving neurological outcomes in adults following cardiac arrest, its role in pediatric patients remains an area of active research and evolving understanding. Ongoing studies focus on determining optimal protocols, including the most effective duration and depth of hypothermia, and identifying specific patient selection criteria for pediatric populations. The challenges in extrapolating findings from adult studies to pediatrics emphasize the need for tailored approaches that consider pediatric patients' unique physiological and developmental aspects. As the evidence base continues to expand, therapeutic hypothermia holds promise as a neuroprotective intervention in pediatric post-resuscitation care, with ongoing efforts to refine its application to maximize benefits for this specific patient population [33].

Emerging Neuroprotective Strategies

Pharmacological interventions: Ongoing research explores various pharmacological agents with potential neuroprotective properties in the post-cardiac arrest phase. These agents include antioxidants, anti-inflammatory drugs, and compounds targeting specific pathways implicated in ischemia-reperfusion injury. The quest for pharmacological interventions seeks to identify substances that can mitigate cellular damage, reduce inflammation, and enhance overall neuroprotection. Exploring pharmacological avenues holds promise for developing novel therapeutic strategies aimed at improving neurological outcomes in pediatric patients following cardiac arrest [34].

Stem cell therapy: Stem cell therapy has emerged as a captivating avenue for neuroprotection in the post-cardiac arrest period. With their regenerative potential and capacity to modulate inflammatory responses, stem cells offer a unique approach to enhancing neurological recovery. Clinical trials are underway to assess stem cell therapy's safety and efficacy in the pediatric population following cardiac arrest. Exploring this innovative therapeutic strategy represents a frontier in neuroprotection research, potentially revolutionizing approaches to post-resuscitation care by harnessing the reparative capabilities of stem cells [35].

Precision medicine approaches: Tailoring neuroprotective strategies based on precision medicine principles is an evolving frontier in post-cardiac arrest care. This approach involves customizing interventions to align with individual patient characteristics, genetic factors, and the specific etiology of cardiac arrest. By considering the unique aspects of each patient's condition, including genetic predispositions and underlying health factors, precision medicine aims to optimize the selection and application of neuroprotective interventions. This personalized approach can enhance the effectiveness of interventions, improve overall outcomes, and pave the way for a more individualized and targeted paradigm in the field of post-resuscitation care for pediatric patients [36].

Cardiovascular care post-resuscitation

Management of Post-Cardiac Arrest Syndrome (PCAS)

Understanding PCAS: PCAS constitutes a complex array of physiological disturbances that manifest after the ROSC. This syndrome encompasses a multifaceted interplay of responses, including a systemic ischemia-reperfusion reaction, myocardial dysfunction, neurological injury, and the lingering risk of persistent factors that precipitate cardiac arrest. A comprehensive understanding of PCAS is foundational for devising targeted interventions to address its diverse components and optimize overall patient recovery [37].

Multifaceted approach: Effectively managing PCAS necessitates a multifaceted and integrated approach. Hemodynamic stabilization is a cornerstone, involving meticulous attention to cardiovascular function to maintain stability. Targeted temperature management, a key component of post-resuscitation care, modulates the body's response and minimizes neurological injury. Respiratory support is equally integral, ensuring optimal oxygenation and ventilation. A coordinated effort to address the distinct facets of PCAS, encompassing cardiovascular, neurological, and respiratory considerations, is crucial. This holistic approach aims to mitigate the impact of PCAS synergistically, enhancing the chances of a favorable outcome for the pediatric patient [38].

Optimizing oxygen delivery: The optimization of oxygen delivery to tissues is central to mitigating the effects of PCAS. This involves a concerted effort to ensure adequate oxygenation, maintain hemodynamic stability, and address potential underlying cardiac arrest causes. By optimizing oxygen delivery, healthcare providers seek to minimize organ dysfunction risk and bolster vital organs' overall resilience post-resuscitation. This proactive strategy encompasses meticulous attention to ventilation parameters, hemodynamic management, and targeted interventions tailored to the specific needs of the pediatric patient. In doing so, the goal is to alleviate the impact of PCAS on organ function and enhance the potential for a positive recovery trajectory [39].

Pharmacological Interventions

Inotropic and vasopressor support: In the presence of persistent myocardial dysfunction and hemodynamic instability post-resuscitation, the administration of inotropic agents, such as dobutamine, and vasopressors, such as epinephrine, becomes a critical component of management. These medications are pivotal in improving cardiac contractility and maintaining systemic blood pressure, supporting adequate organ perfusion. The careful titration of inotropic and vasopressor support is guided by continuous monitoring of hemodynamic parameters, ensuring a tailored approach to address the unique needs of the pediatric patient in the post-cardiac arrest phase [21].

Antiarrhythmic medications: Pharmacological interventions extend to managing arrhythmias that may arise post-resuscitation. Continuous cardiac monitoring guides the administration of antiarrhythmic medications to stabilize cardiac rhythm and prevent further complications. The selection of specific antiarrhythmic agents is based on the type and severity of arrhythmias observed. This proactive approach to rhythm management contributes to the overall stability of the cardiovascular system, minimizing the risk of adverse events related to cardiac dysrhythmias [40].

Volume management: Careful and judicious fluid resuscitation is essential in post-resuscitation care. Balancing fluid administration to optimize preload and cardiac output while avoiding fluid overload is crucial. The intricacies of volume management are guided by continuous assessment of hemodynamic parameters, ensuring that fluid administration aligns with the specific needs of the pediatric patient. This tailored approach helps prevent complications such as pulmonary edema and worsening myocardial function, contributing to the overall stability of the cardiovascular system in the critical post-cardiac arrest phase [41].

Monitoring Cardiac Function

Continuous ECG monitoring: Immediate initiation of continuous ECG monitoring post-resuscitation is a fundamental practice, providing real-time surveillance of cardiac rhythm. This continuous monitoring is essential for promptly detecting any arrhythmias or changes in cardiac rhythm that may arise in the critical post-cardiac arrest phase. The ongoing assessment enables healthcare providers to swiftly intervene, implementing timely adjustments to pharmacological therapies or other interventions to maintain cardiac stability and prevent adverse events [42].

Echocardiography: Bedside echocardiography emerges as a powerful diagnostic tool, offering real-time visualization of cardiac function. This imaging modality allows healthcare providers to assess myocardial contractility, chamber dimensions, and the presence of any structural abnormalities. The dynamic information echocardiography provides is instrumental in tailoring management strategies based on the individual cardiac status of the pediatric patient post-resuscitation. It facilitates a nuanced understanding of cardiovascular dynamics, guiding interventions to optimize cardiac function and mitigate potential complications [43].

Hemodynamic monitoring: Continuous hemodynamic monitoring, encompassing measurements such as CVP and arterial blood pressure, provides valuable insights into the cardiovascular status of the pediatric patient. This information is pivotal in guiding interventions to optimize perfusion, prevent complications, and maintain hemodynamic stability. The continuous nature of hemodynamic monitoring ensures that healthcare providers can promptly respond to changes in the patient's cardiovascular status, contributing to a proactive and tailored approach in post-cardiac arrest care [44].

Biomarkers: Serum biomarkers, including troponin and brain natriuretic peptide (BNP), serve as valuable indicators of myocardial injury and stress. Serial measurements of these biomarkers contribute to the ongoing assessment of cardiac function in the post-resuscitation phase. Elevated levels may signify ongoing cardiac strain or injury, prompting further investigation and guiding therapeutic decision-making. Incorporating biomarkers into the monitoring protocol adds a layer of precision to assessing cardiac status, supporting healthcare providers in optimizing post-resuscitation cardiac care for pediatric patients [45].

Respiratory support and ventilation

Mechanical Ventilation Strategies

Initiation of mechanical ventilation: The initiation of mechanical ventilation is a crucial step in post-cardiac arrest care for many pediatric patients, aiming to ensure optimal oxygenation and ventilation. The decision to initiate mechanical ventilation is a nuanced process influenced by various factors. The patient's level of consciousness, respiratory effort, and the underlying cause of cardiac arrest, all contribute to the determination of whether mechanical support is necessary. Swift and accurate assessment of these factors guides healthcare providers in making informed decisions about the timing and necessity of initiating mechanical ventilation to support respiratory function in the post-resuscitation phase [46].

Ventilator mode selection: The choice of ventilator mode is a personalized decision tailored to each patient's specific needs. Ventilator modes, including volume-controlled ventilation (VCV), pressure-controlled ventilation (PCV), or other hybrid modes, are selected based on a comprehensive evaluation of factors. Lung compliance, airway resistance, and the requirement for synchronized or assist-control ventilation, all contribute to the decision-making process. This tailored approach ensures that the chosen ventilator mode aligns with the patient's respiratory mechanics, optimizing the effectiveness of mechanical support and promoting respiratory stability [47].

Optimizing ventilator settings: Optimizing ventilator settings becomes a continuous and dynamic process once mechanical ventilation is initiated. Adjustments to parameters such as tidal volume, respiratory rate, and positive end-expiratory pressure (PEEP) are meticulously made to achieve adequate gas exchange while minimizing the risk of ventilator-induced lung injury. Lung-protective strategies, including low tidal volume ventilation, are often employed to prevent further damage to lung tissue. The ongoing assessment and refinement of ventilator settings reflect a commitment to providing tailored respiratory support, ensuring that the ventilatory parameters are attuned to the specific needs of the pediatric patient in the critical post-cardiac arrest period [48].

Oxygenation and Ventilation Targets

Arterial blood gas monitoring: Continuous monitoring of arterial blood gases (ABGs) is a cornerstone in post-cardiac arrest care, providing vital insights into the patient's respiratory and metabolic status. This ongoing assessment guides ventilator setting titration to achieve the desired oxygenation and ventilation targets. ABG analysis informs adjustments to inspired oxygen concentration and ventilator parameters, ensuring the maintenance of appropriate arterial oxygen and carbon dioxide levels. This real-time feedback loop, facilitated by ABG monitoring, enables healthcare providers to tailor respiratory support to the dynamic needs of the pediatric patient, optimizing gas exchange and respiratory function [49].

Oxygenation targets: Individualized oxygenation targets are established based on a comprehensive assessment of the patient's clinical condition and underlying pathology. Monitoring oxygen saturation (SpO2) becomes instrumental in achieving these targets, guiding adjustments to inspired oxygen concentrations. The delicate balance lies in optimizing oxygen delivery to tissues while avoiding the potential harm of hyperoxia, including oxidative stress. By closely monitoring oxygenation targets, healthcare providers strive to create an environment that supports tissue oxygenation and minimizes the risk of complications related to imbalances in oxygen levels post-cardiac arrest [50].

Ventilation targets: Ventilation targets revolve around optimizing the elimination of carbon dioxide while preventing excessive ventilation that could lead to respiratory alkalosis. Complementing ABG analysis, end-tidal carbon dioxide (EtCO2) monitoring becomes a valuable tool in assessing ventilation adequacy. This monitoring assists healthcare providers in gauging the patient's ventilatory status and guides adjustments to ventilator settings. The goal is to strike a precise balance, ensuring effective carbon dioxide removal while avoiding the adverse effects of excessive ventilation. By incorporating EtCO2 monitoring into the comprehensive respiratory assessment, healthcare providers can refine ventilator management strategies to meet the specific needs of pediatric patients in the critical post-cardiac arrest phase [51].

Addressing Respiratory Complications

Prevention of ventilator-associated complications: Mitigating the risk of ventilator-associated complications, notably ventilator-associated pneumonia (VAP) and barotrauma, involves implementing a comprehensive set of preventive strategies. Rigorous adherence to proper hand hygiene practices, elevation of the head of the bed to reduce aspiration risk, and meticulous adherence to aseptic techniques during airway management contribute to a multifaceted approach. Collectively, these preventive measures aim to create an environment that minimizes the potential for complications associated with mechanical ventilation, supporting the overall respiratory well-being of the pediatric patient in the post-cardiac arrest phase [52].

Early recognition of respiratory distress: Vigilant monitoring for signs of respiratory distress assumes paramount importance in post-cardiac arrest care. Early recognition of indicators such as increased breathing work, chest retractions, or persistent hypoxemia allows for timely intervention. Healthcare providers can swiftly respond by adjusting to ventilator settings, administering bronchodilators as needed, or considering advanced respiratory support. This proactive approach to recognizing and addressing respiratory distress optimizes respiratory function and mitigates potential complications, fostering a responsive and patient-centered respiratory care model [53].

Pulmonary imaging and diagnostic modalities: Pulmonary imaging, including chest X-rays, is instrumental in assessing lung parenchyma and identifying potential complications. Beyond traditional radiographic methods, advanced diagnostic modalities such as lung ultrasound may be employed to evaluate lung aeration and detect pleural or pulmonary pathology. The integration of these diagnostic tools enhances the precision of respiratory assessment, enabling healthcare providers to promptly identify and address pulmonary issues that may impact the post-resuscitation respiratory status of pediatric patients [54].

Bronchoscopy and airway clearance: In situations where airway obstruction or excessive secretions are suspected, bronchoscopy is a valuable diagnostic and therapeutic tool. Bronchoscopy enables healthcare providers to assess and address airway-related concerns by directly visualizing the airways. Additionally, airway clearance techniques, including chest physiotherapy and mucolytic agents, are employed to maintain airway patency and optimize respiratory function. This proactive approach to airway management contributes to preventing complications and promoting respiratory well-being in pediatric patients post-cardiac arrest [55].

Hemodynamic management

Fluid Resuscitation

Early fluid resuscitation: In the immediate aftermath of resuscitation, initiating early fluid resuscitation is a pivotal component of post-cardiac arrest care. The primary objective is to restore intravascular volume and enhance perfusion swiftly. The choice between crystalloid and colloid solutions is influenced by a nuanced consideration of factors such as the patient's hemodynamic status, electrolyte balance, and specific clinical considerations. Early fluid resuscitation sets the foundation for stabilizing cardiovascular function and supporting vital organ perfusion during the critical post-resuscitation phase [56].

Assessment of fluid responsiveness: The ongoing assessment of fluid responsiveness is a dynamic process that informs continued fluid management. Careful monitoring, including dynamic indicators like changes in pulse pressure or stroke volume in response to fluid administration, is crucial in determining the patient's fluid needs. These indicators provide real-time insights into the patient's cardiovascular responsiveness to fluid, allowing healthcare providers to tailor fluid administration to the individual requirements of the pediatric patient post-cardiac arrest [57].

Caution in fluid administration: While fluid resuscitation is imperative, a judicious and cautious approach is essential to prevent potential complications. These complications may include fluid overload, pulmonary edema, and impaired cardiac function. Regular reassessment of the patient's hemodynamic status becomes paramount, allowing healthcare providers to adjust fluid administration based on the evolving clinical picture. This vigilant and adaptive approach maintains optimal fluid balance, ensuring that the benefits of fluid resuscitation are realized without exposing the patient to unnecessary risks [58].

Inotropic and Vasopressor Support

Indications for inotropic support: In the post-cardiac arrest phase, indications for inotropic support arise when there is evidence of inadequate cardiac output or myocardial dysfunction. Inotropic agents, such as dobutamine, enhance myocardial contractility, thereby improving the heart's pumping efficiency. By augmenting cardiac output, these agents contribute to the overall goal of supporting systemic perfusion, ensuring that vital organs receive an adequate supply of oxygenated blood. The decision to initiate inotropic support is informed by a comprehensive assessment of the patient's cardiovascular function and may be a crucial intervention in optimizing post-resuscitation hemodynamics [59].

Vasopressor support for hemodynamic stability: Vasopressors, including epinephrine and norepinephrine, are critical in maintaining hemodynamic stability post-resuscitation. Indicated in hypotension or inadequate perfusion, these medications exert their effects by increasing systemic vascular resistance and elevating blood pressure. By enhancing vascular tone, vasopressors ensure adequate perfusion to vital organs, mitigating the risk of systemic hypoperfusion. Vasopressor support is a targeted intervention to address specific hemodynamic challenges and stabilize the patient's overall cardiovascular status [60].

Titration of medications: Inotropic and vasopressor medications are a nuanced and individualized process based on the patient's response and ongoing hemodynamic assessment. Continuous monitoring of blood pressure, heart rate, and other relevant parameters forms the basis for the titration process. Healthcare providers carefully adjust medication dosages to achieve the desired hemodynamic goals while minimizing the risk of adverse effects. This dynamic and responsive approach ensures that inotropic and vasopressor support is tailored to the unique needs of the pediatric patient in the post-cardiac arrest phase, optimizing cardiovascular function and promoting hemodynamic stability [60].

Goal-Directed Therapy

Definition and objectives: Goal-directed therapy is a strategic approach in post-cardiac arrest care that involves tailoring interventions to achieve specific hemodynamic goals. These goals typically encompass maintaining a target mean arterial pressure (MAP), ensuring adequate cardiac output, and optimizing oxygen delivery to vital tissues. The overarching objectives of goal-directed therapy are to enhance organ perfusion and prevent complications associated with inadequate perfusion. By setting and pursuing these specific hemodynamic targets, healthcare providers aim to support the recovery of vital organ systems and improve overall patient outcomes [61].

Hemodynamic monitoring: Continuous hemodynamic monitoring is central to the success of goal-directed therapy. Invasive monitoring methods, including arterial blood pressure monitoring and CVP measurement, provide real-time data that guide the implementation of goal-directed interventions. Non-invasive monitoring techniques, such as serial clinical assessments and echocardiography, complement invasive measures, offering a comprehensive perspective on the patient's cardiovascular status. This multi-faceted approach to hemodynamic monitoring ensures that healthcare providers have a dynamic and detailed understanding of the pediatric patient's hemodynamic profile, facilitating precise and targeted goal-directed therapy [62].

Individualized targets: Goal-directed therapy recognizes the importance of individualization in setting hemodynamic targets. The specific goals are tailored based on the patient's age, underlying medical conditions, and the nature of the cardiac arrest. This individualized approach acknowledges each pediatric patient's unique physiological characteristics and needs, allowing targeted therapy to address specific challenges. By aligning hemodynamic goals with the patient's clinical context, goal-directed therapy becomes a personalized strategy to prevent end-organ dysfunction and optimize tissue perfusion [63].

Adaptability to changing conditions: A defining characteristic of goal-directed therapy is its adaptability to changing clinical conditions. The dynamic nature of post-resuscitation care necessitates regular reassessment and adjustment of therapeutic interventions. This ensures that hemodynamic management remains responsive to the evolving needs of the pediatric patient. Adapting real-time interventions enhances goal-directed therapy's efficacy, promoting optimal cardiovascular function and resilience in the face of the complex challenges posed by post-cardiac arrest physiology [64].

Post-resuscitation monitoring

Continuous Monitoring Parameters

Vital signs: Continuous monitoring of vital signs, including heart rate, blood pressure, respiratory rate, and oxygen saturation, is a cornerstone in post-cardiac arrest care. This real-time assessment offers critical insights into the patient's physiological stability. Monitoring trends in these parameters enables healthcare providers to promptly identify and respond to changes, guiding adjustments to therapeutic interventions. Vital sign monitoring is instrumental in gauging the patient's response to treatment, detecting potential complications, and ensuring ongoing physiological homeostasis [65].

ECG: Continuous ECG monitoring is indispensable for detecting a spectrum of cardiac abnormalities in the post-cardiac arrest phase. This includes arrhythmias, conduction abnormalities, and myocardial ischemia or injury signs. The continuous surveillance of the ECG ensures the timely recognition of cardiac rhythm disturbances, allowing healthcare providers to intervene promptly. This vigilance is crucial in post-resuscitation care, where cardiac stability is paramount. ECG monitoring provides a continuous window into the patient's cardiac activity, guiding therapeutic decisions and contributing to the overall cardiovascular assessment [66].

Continuous pulse oximetry: Monitoring oxygen saturation through continuous pulse oximetry is vital for assessing respiratory function in post-cardiac arrest patients. This non-invasive method offers real-time feedback on the patient's oxygenation status. Continuous pulse oximetry is particularly important in guiding adjustments to ventilator settings or oxygen supplementation, ensuring oxygen delivery meets the patient's evolving respiratory needs. This ongoing assessment supports optimizing respiratory parameters, contributing to overall respiratory well-being post-resuscitation [67].

EtCO2: Continuous monitoring of EtCO2 levels provides valuable information about the adequacy of ventilation and perfusion. EtCO2 levels can signal respiratory or circulatory function alterations, prompting further investigation and intervention. In the post-cardiac arrest phase, EtCO2 monitoring is particularly relevant for assessing ventilation effectiveness, guiding adjustments to ventilator settings, and contributing to the early detection of potential respiratory compromise. This parameter is a critical component of the comprehensive respiratory assessment in post-resuscitation care [68].

Invasive hemodynamic monitoring: In cases where close hemodynamic assessment is warranted, invasive monitoring through arterial lines and central venous catheters offers continuous measurement of blood pressure, CVP, and other hemodynamic parameters. This direct and real-time data is invaluable for tailoring interventions to patients' cardiovascular needs. Invasive hemodynamic monitoring provides a more detailed picture of the patient's circulatory status, optimizing fluid management, vasopressor support, and overall hemodynamic stability in the critical post-cardiac arrest period [62].

Biomarkers for Prognostication

Troponin: Serum troponin levels play a crucial role as biomarkers in assessing myocardial injury post-resuscitation. Troponin is specific to cardiac muscle, and elevated blood levels indicate damage to the heart muscle. Monitoring troponin levels provides valuable insights into the extent of cardiac involvement and ongoing stress on the myocardium. Elevated troponin levels may prompt interventions to optimize cardiac function, guiding the management of post-cardiac arrest patients to prevent further cardiac complications [69].

BNP: BNP levels indicate cardiac strain, particularly in assessing myocardial dysfunction. As the heart experiences increased pressure or volume overload, BNP is released. Serial measurements of BNP assist in monitoring the response to treatment and provide valuable information for predicting outcomes. Monitoring BNP levels in the post-resuscitation phase helps healthcare providers assess the cardiac component of post-cardiac arrest syndrome, guiding interventions to mitigate cardiac stress and optimize overall cardiovascular function [70].

S100B protein: S100B is a biomarker associated with neurological injury. Elevated levels of S100B in the bloodstream may suggest ongoing brain damage. Monitoring S100B levels aids in the early identification of neurological complications, allowing for timely intervention and management. This biomarker provides valuable information about the extent of neurological injury post-resuscitation, guiding healthcare providers in tailoring neuroprotective strategies and monitoring the neurological well-being of the pediatric patient [71].

Lactate: Lactate is a critical biomarker that reflects tissue perfusion and metabolic stress. Persistent elevation of lactate levels may indicate inadequate tissue perfusion, suggesting ongoing challenges in meeting metabolic demands. Regular monitoring of lactate levels is instrumental in guiding interventions to improve organ perfusion. Elevated lactate levels prompt healthcare providers to assess and address factors contributing to impaired tissue perfusion. This ensures timely interventions to optimize hemodynamic stability and prevent complications related to inadequate oxygen delivery to tissues [72].

Imaging Studies in the Post-Resuscitation Phase

Chest X-rays: Post-resuscitation chest X-rays are pivotal in assessing pulmonary status and guiding respiratory management. These imaging studies provide valuable information about lung aeration, the correct placement of endotracheal tubes, and the presence of any pulmonary pathology. Changes observed in lung fields on chest X-rays may prompt adjustments to ventilator settings, ensuring optimal respiratory support. This diagnostic tool is instrumental in monitoring lung function and addressing potential complications related to respiratory dynamics in the post-cardiac arrest phase [73].

Echocardiography: Bedside echocardiography is a dynamic and real-time imaging tool employed to assess cardiac function post-cardiac arrest. This modality allows for the evaluation of myocardial contractility, chamber dimensions, and the presence of structural abnormalities. The information obtained from echocardiography guides ongoing hemodynamic management, providing critical insights into the cardiovascular status of the pediatric patient. By visualizing the heart in action, healthcare providers can tailor interventions to optimize cardiac function, contributing to overall hemodynamic stability [74].

Brain imaging: Neuroimaging studies, such as CT or MRI, may be considered in cases where neurological complications are suspected. These studies are crucial in identifying intracranial pathology, including potential signs of brain injury or other neurological issues. The results of brain imaging studies guide healthcare providers in making informed decisions about managing neurological complications, enabling targeted interventions to optimize brain health in the post-resuscitation phase [75].

Abdominal imaging: In selected cases, abdominal imaging studies, such as ultrasound or CT scans, may assess organ perfusion, detect potential abdominal complications, or guide interventions related to the underlying cause of cardiac arrest. These imaging modalities offer valuable information about the condition of abdominal organs, aiding healthcare providers in understanding and addressing potential complications that may impact overall patient stability. Abdominal imaging studies contribute to a comprehensive assessment, allowing for tailored interventions based on the individual patient's specific needs [76].

Long-term outcomes and rehabilitation

Functional Outcomes in Survivors

Neurological function: Assessing neurological function is critical in understanding the long-term outcomes of pediatric patients who have experienced cardiac arrest. Survivors may exhibit a spectrum of neurological sequelae, ranging from minimal impairment to significant deficits. Functional assessments, such as the GOS and the Pediatric Cerebral Performance Category (PCPC), play a crucial role in characterizing the extent of neurological recovery. The GOS evaluates overall outcomes, while the PCPC specifically focuses on neurological performance. These assessments provide healthcare providers with a comprehensive understanding of the impact of cardiac arrest on neurological function and guide interventions to support optimal recovery [77].

Motor function: Motor function is crucial to functional outcomes in post-cardiac arrest survivors. Physical therapy interventions optimize motor skills, mobility, and coordination. The assessment of motor function involves evaluating gross and fine motor abilities, adaptive behaviors, and activities of daily living. By systematically assessing motor function, healthcare providers can tailor rehabilitation strategies to address specific motor challenges and promote the highest level of independence and functionality for pediatric patients in the post-resuscitation phase [78].

Quality of life measures: Long-term outcomes extend beyond immediate medical considerations to encompass the overall quality of life for pediatric cardiac arrest survivors. Patient-reported outcome measures (PROMs) and assessments of health-related quality of life (HRQoL) provide valuable insights into recovery's psychosocial and emotional aspects. These measures capture survivors' subjective experiences and perspectives, allowing healthcare providers to understand the impact of cardiac arrest on various aspects of daily life. Assessing quality of life helps guide interventions that address medical needs and support the broader well-being and satisfaction of pediatric patients and their families throughout the recovery process [79].

Cognitive and Developmental Considerations

Cognitive function: Pediatric cardiac arrest survivors may encounter challenges related to cognitive function, emphasizing the importance of thorough neuropsychological assessments. These assessments help identify deficits in specific cognitive domains such as attention, memory, executive function, and academic performance. Understanding the nuanced cognitive profile of each survivor allows healthcare providers to tailor interventions, including cognitive rehabilitation programs. These programs aim to mitigate cognitive impairments, support educational attainment, and facilitate a smoother transition back into academic settings. By addressing cognitive challenges, healthcare providers contribute to pediatric patients' overall well-being and potential future success in their cognitive development [80].

Developmental milestones: Given the age of pediatric patients, monitoring developmental milestones is crucial for assessing long-term outcomes. Developmental assessments track progress in language acquisition, socialization, and adaptive skills. Early identification of developmental delays is essential for implementing timely interventions, which may include speech therapy, occupational therapy, and educational support. By addressing developmental needs, healthcare providers contribute to the holistic development of pediatric patients, aiming to enhance their overall functioning and independence as they progress through childhood and adolescence [81].

Psychosocial well-being: The psychosocial impact of cardiac arrest and its aftermath should not be underestimated. Mental health assessments and counseling services are crucial in supporting pediatric patients and their families through the emotional challenges of recovery. Assessments of psychosocial well-being help identify any emotional or psychological distress, allowing for targeted interventions. Counseling services provide a supportive space for patients and their families to navigate the emotional complexities of the post-resuscitation period. By addressing psychosocial needs, healthcare providers contribute to pediatric patients' mental and emotional resilience, fostering a positive trajectory in their overall recovery journey [82].

Rehabilitation Strategies

Physical therapy: Physical therapy is a cornerstone of rehabilitation, focusing on enhancing motor function, strength, and coordination in pediatric cardiac arrest survivors. Individualized rehabilitation plans are tailored to each patient's needs, incorporating various exercises, mobility training, and activities to improve overall physical well-being. Physical therapists work collaboratively with patients to address motor challenges, optimize mobility, and promote physical independence. Through targeted interventions, physical therapy contributes to the restoration of motor skills and the overall functional capacity of pediatric patients, fostering their ability to engage in daily activities [83].

Occupational therapy: Occupational therapy is crucial in addressing daily living skills, fine motor coordination, and adaptive behaviors in pediatric cardiac arrest survivors. Interventions provided by occupational therapists focus on enhancing independence in self-care, school-related tasks, and age-appropriate activities. By targeting specific areas of functional impairment, occupational therapy aims to improve overall occupational performance and quality of life. Incorporating adaptive strategies and skill-building activities supports pediatric patients in achieving greater autonomy and participation in meaningful daily activities [84].

Speech and language therapy: Speech and language therapy is essential for pediatric patients facing communication challenges post-cardiac arrest. This form of therapy encompasses interventions to improve articulation, language comprehension, and social communication skills. Speech and language therapists work with patients to address speech impediments, language deficits, and communication difficulties. The goal is to enhance effective communication, facilitate social interactions, and support academic and social success for pediatric patients on their journey to recovery [85].

Neuropsychological rehabilitation: Neuropsychological rehabilitation addresses cognitive deficits in pediatric cardiac arrest survivors. This form of rehabilitation provides strategies to enhance attention, memory, and executive function. Educational support and accommodations are often integral to neuropsychological rehabilitation, ensuring pediatric patients receive the necessary resources to navigate academic challenges. By tailoring interventions to the specific cognitive needs of each patient, neuropsychological rehabilitation supports the development of cognitive skills. It contributes to the overall cognitive well-being of pediatric cardiac arrest survivors [86].

Family-centered care: In the pediatric population, family-centered care is paramount in the rehabilitation process for cardiac arrest survivors. This approach emphasizes families' involvement in their child's care and recovery. Healthcare providers collaborate closely with families, recognizing them as essential partners in decision-making and goal-setting. By fostering open communication and involving families in the rehabilitation plan, family-centered care creates a supportive and collaborative environment that enhances pediatric cardiac arrest survivors' overall well-being and recovery [87].

School reintegration: Successful school reintegration is a crucial aspect of the rehabilitation process for pediatric cardiac arrest survivors. Collaboration with educational professionals is essential to develop individualized education plans (IEPs) and accommodations that support academic progress while addressing any challenges arising from cognitive or physical impairments. By working closely with schools, healthcare providers ensure a seamless transition back into the educational environment, providing the necessary support for the child to thrive academically and socially [88].

Community-based interventions: Engaging with community resources and support networks is instrumental in promoting the overall well-being of pediatric cardiac arrest survivors. Community-based interventions extend beyond clinical settings and may include recreational therapy, socialization programs, and peer support. These interventions enhance the child's social integration, encourage participation in community activities, and provide a holistic approach to rehabilitation. By tapping into community resources, healthcare providers contribute to the broader support network that plays a vital role in the ongoing recovery and quality of life for pediatric patients and their families [89].

## Conclusions

In conclusion, the comprehensive review of post-resuscitation care in PICUs following cardiac arrest underscores the critical importance of a systematic and multidisciplinary approach. The immediate post-resuscitation phase requires careful navigation, from adherence to resuscitation guidelines and advances in therapeutic interventions to the nuanced management of neurological considerations, cardiovascular care, and respiratory support. Long-term outcomes and rehabilitation strategies are pivotal in optimizing the quality of life for pediatric survivors, emphasizing the need for individualized and family-centered care. A resounding call to action emerges as we reflect on these key findings. Continuous education, research initiatives, and quality improvement efforts are imperative, alongside strengthened multidisciplinary collaboration and advocacy for public awareness. By embracing these principles, the healthcare community can collectively contribute to ongoing advancements in pediatric post-resuscitation care, ultimately improving outcomes and fostering a culture of excellence in pediatric critical care.

## References

[1] Tress EE, Kochanek PM, Saladino RA, Manole MD (2010). Cardiac arrest in children. J Emerg Trauma Shock.

[2] Vega RM, Kaur H, Sasaki J, Edemekong PF (2023). Cardiopulmonary arrest in children. StatPearls [Internet].

[3] Sperotto F, Gearhart A, Hoskote A (2023). Cardiac arrest and cardiopulmonary resuscitation in pediatric patients with cardiac disease: a narrative review. Eur J Pediatr.

[4] Obermaier M, Katzenschlager S, Kofler O, Weilbacher F, Popp E (2022). Advanced and invasive cardiopulmonary resuscitation (CPR) techniques as an adjunct to advanced cardiac life support. J Clin Med.

[5] Lin Y, Cheng A (2015). The role of simulation in teaching pediatric resuscitation: current perspectives. Adv Med Educ Pract.

[6] Mir T, Shafi OM, Uddin M, Nadiger M, Sibghat Tul Llah F, Qureshi WT (2022). Pediatric cardiac arrest outcomes in the United States: a nationwide database cohort study. Cureus.

[7] Blinder J, Nadkarni V, Naim M, Rossano JW, Berg RA (2013). Epidemiology of pediatric cardiac arrest. Pediatric and Congenital Cardiology, Cardiac Surgery and Intensive Care.

[8] Alten JA, Klugman D, Raymond TT (2017). Epidemiology and outcomes of cardiac arrest in pediatric cardiac intensive care units. Pediatr Crit Care Med.

[9] Panchal AR, Bartos JA, Cabañas JG (2020). Part 3: adult basic and advanced life support: 2020 American Heart Association guidelines for cardiopulmonary resuscitation and emergency cardiovascular care. Circulation.

[10] Meaney PA, Bobrow BJ, Mancini ME (2013). Cardiopulmonary resuscitation quality: [corrected] improving cardiac resuscitation outcomes both inside and outside the hospital: a consensus statement from the American Heart Association. Circulation.

[11] Peltonen V, Peltonen LM, Rantanen M (2022). Randomized controlled trial comparing pit crew resuscitation model against standard advanced life support training. J Am Coll Emerg Physicians Open.

[12] Haskell SE, Atkins DL (2010). Defibrillation in children. J Emerg Trauma Shock.

[13] (2015). Strategies to Improve Cardiac Arrest Survival: A Time to Act. https://pubmed.ncbi.nlm.nih.gov/26225413/.

[14] Aaron SL, Vega RM, Hai O (2023). Pediatric postresuscitation management. StatPearls [Internet].

[15] Kumar N, Akangire G, Sullivan B, Fairchild K, Sampath V (2020). Continuous vital sign analysis for predicting and preventing neonatal diseases in the twenty-first century: big data to the forefront. Pediatr Res.

[16] Kang Y (2019). Management of post-cardiac arrest syndrome. Acute Crit Care.

[17] Neatherlin JS, Brillhart B (1988). Glasgow Coma Scale scores in the patient post cardiopulmonary resuscitation. J Neurosci Nurs.

[18] Friedman D, Claassen J, Hirsch LJ (2009). Continuous electroencephalogram monitoring in the intensive care unit. Anesth Analg.

[19] Rivera-Lara L, Zhang J, Muehlschlegel S (2012). Therapeutic hypothermia for acute neurological injuries. Neurotherapeutics.

[20] Marik PE, Monnet X, Teboul JL (2011). Hemodynamic parameters to guide fluid therapy. Ann Intensive Care.

[21] VanValkinburgh D, Kerndt CC, Hashmi MF (2023). Inotropes and vasopressors. StatPearls [Internet].

[22] Virág M, Leiner T, Rottler M, Ocskay K, Molnar Z (2021). Individualized hemodynamic management in sepsis. J Pers Med.

[23] Kneyber MC, de Luca D, Calderini E (2017). Recommendations for mechanical ventilation of critically ill children from the Paediatric Mechanical Ventilation Consensus Conference (PEMVECC). Intensive Care Med.

[24] Kjaergaard J, Schmidt H, Møller JE, Hassager C (2022). The "Blood pressure and oxygenation targets in post resuscitation care, a randomized clinical trial": design and statistical analysis plan. Trials.

[25] Omairi AM, Pandey S (2023). Targeted temperature management. StatPearls [Internet].

[26] Leong SH, Chan E, Ho BC (2017). Therapeutic temperature management (TTM): post-resuscitation care for adult cardiac arrest, with recommendations from the National TTM Workgroup. Singapore Med J.

[27] Soares RO, Losada DM, Jordani MC, Évora P, Castro-E-Silva O (2019). Ischemia/reperfusion injury revisited: an overview of the latest pharmacological strategies. Int J Mol Sci.

[28] Allen KA, Brandon DH (2011). Hypoxic ischemic encephalopathy: pathophysiology and experimental treatments. Newborn Infant Nurs Rev.

[29] Sandroni C, Cronberg T, Sekhon M (2021). Brain injury after cardiac arrest: pathophysiology, treatment, and prognosis. Intensive Care Med.

[30] Sandroni C, Cronberg T, Hofmeijer J (2022). EEG monitoring after cardiac arrest. Intensive Care Med.

[31] Takegawa R, Hayashida K, Rolston DM (2020). Near-infrared spectroscopy assessments of regional cerebral oxygen saturation for the prediction of clinical outcomes in patients with cardiac arrest: a review of clinical impact, evolution, and future directions. Front Med (Lausanne).

[32] Song SS, Lyden PD (2012). Overview of therapeutic hypothermia. Curr Treat Options Neurol.

[33] Colls Garrido C, Riquelme Gallego B, Sánchez García JC, Cortés Martín J, Montiel Troya M, Rodríguez Blanque R (2021). The effect of therapeutic hypothermia after cardiac arrest on the neurological outcome and survival-a systematic review of RCTs published between 2016 and 2020. Int J Environ Res Public Health.

[34] Choudhary RC, Shoaib M, Sohnen S (2021). Pharmacological approach for neuroprotection after cardiac arrest-a narrative review of current therapies and future neuroprotective cocktail. Front Med (Lausanne).

[35] Berlet R, Anthony S, Brooks B (2021). Combination of stem cells and rehabilitation therapies for ischemic stroke. Biomolecules.

[36] Gu S, Luo Q, Wen C (2023). Application of advanced technologies—nanotechnology, genomics technology, and 3D printing technology—in precision anesthesia: a comprehensive narrative review. Pharmaceutics.

[37] Penketh J, Nolan JP (2023). Post-cardiac arrest syndrome. J Neurosurg Anesthesiol.

[38] (2020). 40th International Symposium on Intensive Care & Emergency Medicine: Brussels, Belgium. 24-27 March 2020. Crit Care.

[39] Russell A, Rivers EP, Giri PC, Jaehne AK, Nguyen HB (2020). A physiologic approach to hemodynamic monitoring and optimizing oxygen delivery in shock resuscitation. J Clin Med.

[40] Zimetbaum P (2012). Antiarrhythmic drug therapy for atrial fibrillation. Circulation.

[41] Zhang Y, McCurdy MT, Ludmir J (2023). Sepsis management in the cardiac intensive care unit. J Cardiovasc Dev Dis.

[42] Sattar Y, Chhabra L (2023). Electrocardiogram. StatPearls [Internet].

[43] Keller M, Magunia H, Rosenberger P, Koeppen M (2023). Echocardiography as a tool to assess cardiac function in critical care—a review. Diagnostics (Basel).

[44] Lee EP, Wu HP, Chan OW, Lin JJ, Hsia SH (2022). Hemodynamic monitoring and management of pediatric septic shock. Biomed J.

[45] Chan D, Ng LL (2010). Biomarkers in acute myocardial infarction. BMC Med.

[46] Sutherasan Y, Peñuelas O, Muriel A (2015). Management and outcome of mechanically ventilated patients after cardiac arrest. Crit Care.

[47] Campbell RS, Davis BR (2002). Pressure-controlled versus volume-controlled ventilation: does it matter?. Respir Care.

[48] Mora Carpio AL, Mora JI (2023). Ventilator Management. StatPearls [Internet].

[49] Roupie EE (1997). Continuous assessment of arterial blood gases. Crit Care.

[50] Semler MW, Casey JD, Lloyd BD (2022). Oxygen-saturation targets for critically ill adults receiving mechanical ventilation. N Engl J Med.

[51] Waldmann C, Rhodes A, Soni N, Handy J (2008). Respiratory therapy techniques. Oxford Desk Reference: Critical Care.

[52] Keyt H, Faverio P, Restrepo MI (2014). Prevention of ventilator-associated pneumonia in the intensive care unit: a review of the clinically relevant recent advancements. Indian J Med Res.

[53] Coudroy R, Frat JP, Boissier F, Contou D, Robert R, Thille AW (2018). Early identification of acute respiratory distress syndrome in the absence of positive pressure ventilation: implications for revision of the berlin criteria for acute respiratory distress syndrome. Crit Care Med.

[54] Touw HR, Parlevliet KL, Beerepoot M (2018). Lung ultrasound compared with chest X-ray in diagnosing postoperative pulmonary complications following cardiothoracic surgery: a prospective observational study. Anaesthesia.

[55] Paradis TJ, Dixon J, Tieu BH (2016). The role of bronchoscopy in the diagnosis of airway disease. J Thorac Dis.

[56] Fletcher DJ, Boller M (2020). Fluid therapy during cardiopulmonary resuscitation. Front Vet Sci.

[57] Cherpanath TG, Geerts BF, Lagrand WK, Schultz MJ, Groeneveld AB (2013). Basic concepts of fluid responsiveness. Neth Heart J.

[58] Castera MR, Borhade MB (2023). Fluid management. StatPearls [Internet].

[59] Tariq S, Aronow WS (2015). Use of inotropic agents in treatment of systolic heart failure. Int J Mol Sci.

[60] Pollard S, Edwin SB, Alaniz C (2015). Vasopressor and inotropic management of patients with septic shock. P T.

[61] Lazzarin T, Tonon CR, Martins D (2022). Post-cardiac arrest: mechanisms, management, and future perspectives. J Clin Med.

[62] Huygh J, Peeters Y, Bernards J, Malbrain ML (2016). Hemodynamic monitoring in the critically ill: an overview of current cardiac output monitoring methods. F1000Res.

[63] Kaufmann T, Saugel B, Scheeren TW (2019). Perioperative goal-directed therapy - what is the evidence?. Best Pract Res Clin Anaesthesiol.

[64] Kendrick JB, Kaye AD, Tong Y, Belani K, Urman RD, Hoffman C, Liu H (2019). Goal-directed fluid therapy in the perioperative setting. J Anaesthesiol Clin Pharmacol.

[65] Selvaraju V, Spicher N, Wang J (2022). Continuous monitoring of vital signs using cameras: a systematic review. Sensors (Basel).

[66] Serhani MA, T El Kassabi H, Ismail H, Nujum Navaz A (2020). ECG monitoring systems: review, architecture, processes, and key challenges. Sensors (Basel).

[67] Pandya NK, Sharma S (2023). Capnography and pulse oximetry. StatPearls [Internet].

[68] (1997). Reproductive Health in Developing Countries: Expanding Dimensions, Building Solutions. https://pubmed.ncbi.nlm.nih.gov/25121311/.

[69] Babuin L, Jaffe AS (2005). Troponin: the biomarker of choice for the detection of cardiac injury. CMAJ.

[70] Cao Z, Jia Y, Zhu B (2019). BNP and NT-proBNP as diagnostic biomarkers for cardiac dysfunction in both clinical and forensic medicine. Int J Mol Sci.

[71] Schulte S, Podlog LW, Hamson-Utley JJ, Strathmann FG, Strüder HK (2014). A systematic review of the biomarker S100B: implications for sport-related concussion management. J Athl Train.

[72] Foucher CD, Tubben RE (2023). Lactic acidosis. StatPearls [Internet].

[73] Khan AN, Al-Jahdali H, Al-Ghanem S, Gouda A (2009). Reading chest radiographs in the critically ill (Part I): normal chest radiographic appearance, instrumentation and complications from instrumentation. Ann Thorac Med.

[74] Esmaeilzadeh M, Parsaee M, Maleki M (2013). The role of echocardiography in coronary artery disease and acute myocardial infarction. J Tehran Heart Cent.

[75] Corrêa DG, de Souza SR, Nunes PG, Coutinho AC Jr, da Cruz LC Jr (2022). The role of neuroimaging in the determination of brain death. Radiol Bras.

[76] Caraiani C, Yi D, Petresc B, Dietrich C (2020). Indications for abdominal imaging: when and what to choose?. J Ultrason.

[77] Huebschmann NA, Cook NE, Murphy S, Iverson GL (2022). Cognitive and psychological outcomes following pediatric cardiac arrest. Front Pediatr.

[78] Combs S, Miller EW, Forsyth E (2007). Motor and functional outcomes of a patient post-stroke following combined activity and impairment level training. Physiother Theory Pract.

[79] Haywood KL, Pearson N, Morrison LJ, Castrén M, Lilja G, Perkins GD (2018). Assessing health-related quality of life (HRQoL) in survivors of out-of-hospital cardiac arrest: a systematic review of patient-reported outcome measures. Resuscitation.

[80] Kim SH, Oh SH, Park KN, Kim TH (2019). Cognitive impairment among cardiac arrest survivors in the ICU: a retrospective study. Emerg Med Int.

[81] Choo YY, Agarwal P, How CH, Yeleswarapu SP (2019). Developmental delay: identification and management at primary care level. Singapore Med J.

[82] Agarwal S, Birk JL, Abukhadra SL (2022). Psychological distress after sudden cardiac arrest and its impact on recovery. Curr Cardiol Rep.

[83] Ward KM, Wittekind SG, White DA (2023). Pediatric physical activity promotion, exercise therapy and cardiac rehabilitation. Pediatric Cardiology.

[84] (2023). Occupational Therapy (for Parents). https://kidshealth.org/en/parents/occupational-therapy.html.

[85] Pennington L, Goldbart J, Marshall J (2004). Speech and language therapy to improve the communication skills of children with cerebral palsy. Cochrane Database Syst Rev.

[86] Ubeda Tikkanen A, Vova J, Holman L (2023). Core components of a rehabilitation program in pediatric cardiac disease. Front Pediatr.

[87] Kuo DZ, Houtrow AJ, Arango P, Kuhlthau KA, Simmons JM, Neff JM (2012). Family-centered care: current applications and future directions in pediatric health care. Matern Child Health J.

[88] Blakeney P (1995). School reintegration. J Burn Care Rehabil.

[89] Simmons KM, McIsaac SM, Ohle R (2023). Impact of community-based interventions on out-of-hospital cardiac arrest outcomes: a systematic review and meta-analysis. Sci Rep.

